# Treatment of cervical vertebral (C1) metastasis of lung cancer with radiotherapy: A case report

**DOI:** 10.3892/ol.2013.1183

**Published:** 2013-02-06

**Authors:** XUEFENG NI, PING WU, CHANGPING WU, JIANFENG WU, MEI JI, XIAOFANG GU, BO TIAN

**Affiliations:** 1 Departments of Oncology, The Third Affiliated Hospital, Soochow University, Changzhou, Jiangsu 213003;; 2 Pharmacology, The Third Affiliated Hospital, Soochow University, Changzhou, Jiangsu 213003;; 3 Department of Radiation Oncology, Jiangsu Cancer Hospital and Research Institute, Nanjing, Jiangsu 210009;; 4 Departments of Radiology, The Third Affiliated Hospital, Soochow University, Changzhou, Jiangsu 213003, P.R. China; 5 Pathology, The Third Affiliated Hospital, Soochow University, Changzhou, Jiangsu 213003, P.R. China

**Keywords:** metastasis, cervical vertebrae, radiotherapy

## Abstract

The present study discusses a patient with C1 vertebral metastasis from adenocarcinoma of the left lung. The patient was a 31-year-old female suffering from neck pain who was referred by her physician. Magnetic resonance imaging revealed osteolytic destruction of the C1 vertebra. Chest and computed tomographic scans revealed lung carcinoma changes involving the left lung. A biopsy confirmed adenocarcinoma of the left lung. Abnormal activity was present in the cervical spine (C1) region in a radionuclide bone scan. The patient was then referred to an oncologist. The spine was stabilized with a rigid collar and a course of radiation therapy and pain medication was initiated immediately. At the 9-month follow-up examination, there was no evidence of progression on the MRI scans and the main neck symptoms had disappeared. At present, the overall survival (OS) time is 11 months. Patients complaining of new onset back or neck pain should be assumed to have vertebral metastasis until proven otherwise. Trivial trauma should be taken seriously in these cases and investigated with appropriate clinical, laboratory and imaging examinations.

## Introduction

The spine is the most common site of skeletal metastases, with >18,000 new cases of spinal metastases recognized annually (1,2). Between 5 and 10% of patients with systemic cancer develop vertebral metastases (3–6). The thoracic spine is the most common region involved in spinal metastases (70%), followed by the lumbar spine (20%), while the cervical region is affected in 10% of cases (7). Lung, prostate, breast, renal cell, thyroid and gastrointestinal carcinomas are the most common tumors that metastasize to the spinal column (4,8–10). The most common symptom in cervical metastases is neck pain which occurs in 90% of patients; 50% of cases complain of severe deficits, such as acute weakness that may progress to quadriplegia (11–13). The median survival time after the first detection of skeletal metastasis is 3–6 months in squamous cell lung carcinoma, 20 months in breast carcinoma and 40 months in prostate carcinoma (14).

Metastatic destruction of the vertebral bodies may result in pathological compression fractures, leading to angulated kyphotic deformities that may be observed clinically or in imaging studies (15,16). The upper cervical spine has the largest spinal canal and therefore neurological symptoms typically result from instability rather than compressive insult (16). The occipitoatlantoaxial spine is rarely affected, particularly the C1 vertebra.

The majority of vertebral metastases originate via hematogenous dissemination from primary carcinomas of the breast, lung or prostate (17). In the osteolytic form of vertebral metastasis, tumor cells infiltrate the trabecular matrix of the bone, resulting in a loss of osseous integrity, predisposing the spine to pathological fractures (14).

Radiotherapy (RT) is important in palliating the symptoms of patients with metastatic disease. RT techniques are used in a broad range of circumstances, including as a prophylactic measure against future pathological fractures and palliation of bone pain, as well as severe symptoms associated with cord compression and impending neurological compromise.

The beneficial effect of achieving analgesia of bone metastases with RT is well documented. The response to RT has been quantified and qualified with numerous criteria and instruments over the past decades. Additionally, evidence reveals that 70–90% of patients achieve a beneficial response due to analgesic-directed RT with complete responses observed in up to 40% of patients (18–20).

Modern advances in computer technology and the delivery of RT have led to the development of treatment techniques, such as 3-dimensional (3D) conformal, intensity-modulated and proton beam therapy. However, since the majority of spinal tumors are metastases, spinal RT is often delivered using conventional 2-dimensional (2D) or 3D conformal techniques.

## Case report

The patient was a 31-year-old female suffering from neck pain for 1 month prior to the discovery of a mass in the neck. A physical examination revealed a tender mass and motion in the cervical vertebrae was limited. At the time of examination, the patient’s neck pain was constant in the suboccipital region, rated at 3–4 out of 10 on a pain scale. Upon any movement, however, the ache became a sharp pain, rated at 7–8 out of 10. The patient had no history of neck trauma. Initial lateral and anteroposterior open-mouth cervical spine radiographs and computed tomography (CT) of the cervical spine were obtained when the patient first experienced neck pain. The CT revealed osteolytic destruction involving the C1 vertebra (images not shown).

**Figure 1:**
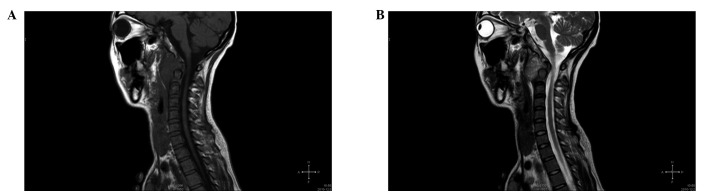
Female, 31-year-old patient with severe neck pain. Sagittal MRI scans revealed an extremely large chordoma centered at C1 and extending to the anterior aspects of C2. (A) T1-weighted and (B) T2-weighted sagittal MRI. MRI, magnetic resonance imaging.

Magnetic resonance imaging (MRI) scans revealed an extremely large tumor centered on the C1 vertebra, as well as a soft tissue mass beside the C1 vertebra, which extended into the anterior aspects of C2 (Fig. 1). MRI is the most sensitive test available for the evaluation of the soft tissue extent of the tumor. The MRI appearance was nonspecific, with T1-weighted images showing a low signal (Fig. 1A) and T2-weighted images showing an intermediate-to-high signal within the mass (Fig. 1B).

**Figure 2:**
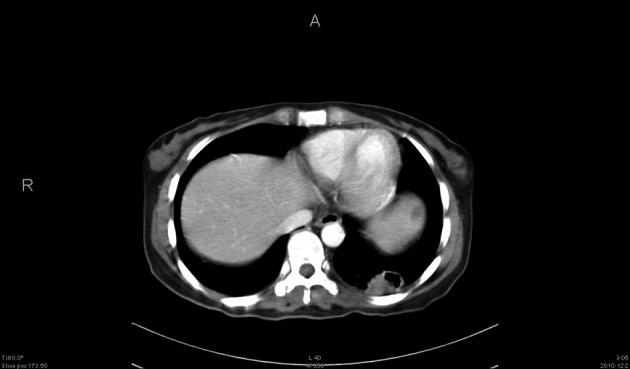
Initial transaxial computed tomographic scan showing a mass in the left lung.

**Figure 3:**
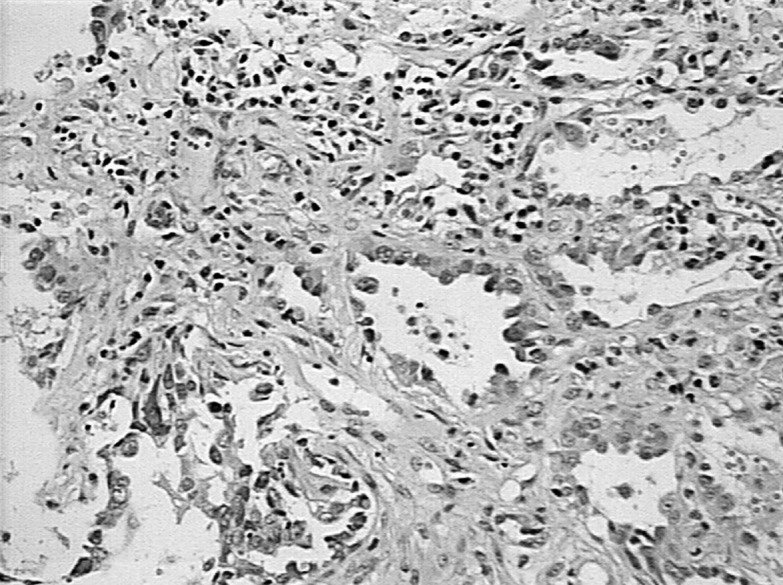
Paraffin section of a biopsy obtained via bronchoscopy stained with HE, revealing adenocarcinoma of the left lung. HE, hematoxylin and eosin.

Chest and CT scans then revealed lung carcinoma changes involving the left lung (Fig. 2). A paraffinized section of a biopsy obtained via bronchoscopy was stained with HE. The biopsy confirmed adenocarcinoma of the left lung (Fig. 3).

**Figure 4:**
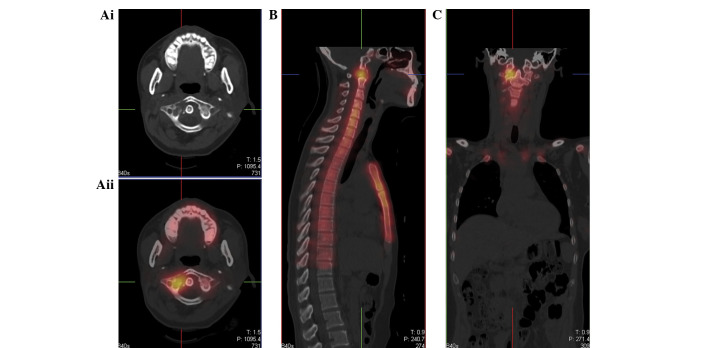
(Ai) Axial CT scan showing osteolytic destruction of the C1 vertebra. Radionuclide bone scan after injection of 24 mCi of ^99m^TC-MDP showing (Aii) the axial view of the cervical spine, (B) sagittal image of the cervical spine and (C) coronal image of the cervical spine. Abnormal activity was present in the C1 region (yellow region in Aii, B and C). These areas of increased radiopharmaceutical uptake represent metastatic skeletal deposits. CT, computed tomography.

Axial CT scanning showed evident osteolytic destruction of the C1 vertebra (Fig. 4Ai). A whole-body radionuclide bone scan exhibited increased pharmaceutical uptake in the region of the known lesion in the upper cervical spine (Fig. 4Aii, B and C), as well as the proximal portion of the left femur (images not shown), consistent with further metastatic infiltration. The patient was then fitted with a rigid cervical spine brace in an attempt to stabilize the spine and limit cord compression.

Neurosurgeons were consulted and declined to intervene due to the poor overall prognosis and the patient elected to avoid aggressive treatment. A management plan, consisting of radiation therapy on the cervical spine, analgesic medication and close monitoring with radiography and advanced imaging, was initiated.

**Figure 5:**
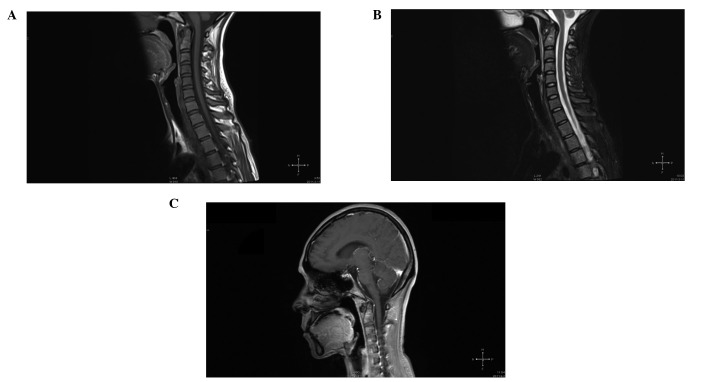
Follow-up cervical spine MRI obtained at 3 months after radiotherapy; (A) T1-weighted and (B) T2-weighted sagittal MRI. (C) Follow-up cervical spine MRI obtained at 9 months after radiotherapy showing permeative stablility of C1; T1-weighted Sagittal MRI. MRI, magnetic resonance imaging.

The patient agreed to proceed with radiation therapy for the disease after the first MRI scan. The schedule was conventional RT with 5 daily 4-Gy fractions. After RT, the patient felt that the pain had been significantly relieved and reported no swallowing dysfunction. Chemotherapy was administered after the RT. Subsequent follow-up MRI scans at 3 (Fig. 5A and B) and 9 months (Fig. 5C) after RT revealed no progression of the osseous destruction. At 3 months after RT, the rigid cervical spine brace was removed from the patient. At present, the overall survival (OS) time is 11 months. At the 9-month follow-up examination, the main neck symptoms had disappeared.

## Discussion

The present study describes the case of a patient undergoing cervical spine radiation therapy for a known lung cancer who was referred to a neck pain department.

Spinal symptoms are the first indication of skeletal metastasis in 20% of cancer patients (15). The most common clinical feature in a patients with vertebral metastases is pain, although neurological symptoms may also be present (17). Trivial trauma should be taken seriously in patients with cancer and evaluated with appropriate diagnostic imaging.

The most widely available imaging modality is conventional radiography. However, bone scans are relatively nonspecific and present difficulties in differentiating between infection, fracture, spondylosis and tumors (16). MRI is sensitive and specific and has become the gold standard for evaluating vertebral metastases (17,21,22). MRI is extremely sensitive to pathological changes in bone marrow, as well as the detection of cord and nerve compression (17).

Prior to RT, cervical spine biopsies had not been obtained from the patient since biopsies of the cervical spine have significant risks as the tumor is surrounded by the vertebral artery, spinal cord and nerve root.

In general, the treatment of spinal tumors is surgical and en bloc resection with negative margins has been shown to decrease the rates of local and metastatic recurrence (23–26). En bloc resection is now the standard of care for numerous primary tumors of the thoracic, lumbar and sacral spine. However, several factors complicate the performing of this procedure in the cervical spine, including the proximity of the vertebral arteries, intricate bony architecture and importance of the cervical nerve roots. Furthermore, en bloc excision of cervical spinal tumors involves long operative times and significant perioperative morbidity.

It is difficult to remove tumors en bloc from the cervical spine and there is a high rate of recurrence and metastasis. Due to these factors, as well as the relative rarity of cases, this technique has not been widely adopted in the cervical spine.

RT may be used in place of surgery, serve as an adjunct to surgery or as a preparative regimen to make a tumor more readily resectable. RT in the complicated and uncomplicated spinal metastasis setting is commonly prescribed for the posterior wall of the vertebral body (or anterior aspect of the spinal cord proper). There is no standard treatment approach insofar as there are numerous dose fractionation schedules for uncomplicated spinal skeletal metastasis.

The majority of practitioners prefer a more protracted course of RT in cases of cord compression and courses vary from 5 daily fractions of 4 Gy to 23 daily fractions of 2 Gy (19). In the USA, the most common schedule is 10 daily 3-Gy fractions. A number of study series included patients treated with a single 8-Gy fraction course and no significant difference was observed in the clinical outcomes or late toxicity (27,28). However, the available data appear to indicate no significant benefits of one fractionation schedule over another when analyzing the functional outcomes (27–30).

Patients complaining of new onset back or neck pain should be assumed to have vertebral metastasis until proven otherwise. Trivial trauma should be taken seriously in these cases and investigated with appropriate clinical, laboratory and imaging examinations.

However, RT should be used with caution, as the spinal cord is sensitive to radiation; local irradiation is suggested. A schedule of 5 daily 4-Gy fractions was successful for the present patient. In MRI scans, there was a nearly complete response in the C1 vertebra.
